# Dutch rehabilitation physicians’ perspectives on contracture management in children with spinal muscular atrophy: challenges in a changing landscape

**DOI:** 10.3389/fneur.2025.1670391

**Published:** 2025-12-10

**Authors:** Irene L. B. Oude Lansink, Anita Beelen, Esther Kruitwagen-van Reenen, Sanne van der Vossen, Jan Willem Gorter

**Affiliations:** 1 Department of Rehabilitation, Physical Therapy Science and Sports, University Medical Center Utrecht, Wilhelmina Children's Hospital, Utrecht, Netherlands; 2 Center of Excellence for Rehabilitation Medicine, UMC Utrecht Brain Center, University Medical Center Utrecht, De Hoogstraat Rehabilitation, Utrecht, Netherlands; 3 Department of Child and Youth, De Hoogstraat Rehabilitation, Utrecht, Netherlands; 4 Department of Pediatrics, CanChild Center for Childhood Disability Research, McMaster University, Hamilton, ON, Canada

**Keywords:** spinal muscular atrophy, contractures, treatment, children, adolescents

## Abstract

**Background:**

Many children with hereditary proximal spinal muscular atrophy (SMA) develop joint contractures. With the introduction of disease-modifying treatments (DMTs) for SMA, the improved functional prognosis may change the focus of (preventive) contracture management.

**Objective:**

This study aimed to describe current approaches to contracture management among Dutch pediatric rehabilitation physicians caring for children with SMA receiving DMT, and to explore the underlying considerations and clinical reasoning that inform their decisions on contracture management in the evolving therapeutic landscape.

**Methods:**

All registered pediatric rehabilitation physicians (n = 151) received a survey, addressing two main topics: (1) indication and purpose of contracture management, and (2) alignment of clinical decision-making with current guidelines. To this end, three standardized case scenarios were presented. Respondents were asked to indicate whether their current choices, were consistent with the guideline recommendations. To obtain a deeper understanding of the considerations and clinical reasoning regarding contracture management in the era of DMTs, we held an advisory group meeting. We audio-recorded the discussions and analyzed the content thematically.

**Results:**

The response rate was 56%; 41 of these respondents were not involved in SMA care. 38 of the 44 surveys, completed by participants involved in SMA care, were suitable for analysis. All respondents (strongly) agreed about ‘optimal sitting posture’ being an important treatment goal, 95% agreed on ‘pain prevention’ and 87% on ‘maintaining function’. Physicians recommended daily use of hand splints less frequently in children who started DMT before onset of symptoms (35%) than in children who started DMT at an advanced disease stage (54%). Thematic analysis revealed three themes shaping clinical reasoning: (1) functional prognosis as key element in decision-making; (2) clinical uncertainty regarding contracture intervention; and (3) incorporation of contextual factors.

**Conclusion:**

Dutch pediatric rehabilitation physicians describe challenges in clinical decision-making regarding contracture management in a changing landscape for SMA. The use of key principles could facilitate the process, including: (1) assessing the child’s functional prognosis; (2) engaging in open discussions with parents about uncertainties arising from limited clinical experience and the evolving understanding of disease trajectories in the early post-DMT era; and (3) applying the ICF framework to incorporate contextual factors into clinical decision-making regarding contracture management.

## 1 Introduction

Hereditary proximal spinal muscular atrophy (SMA) is an autosomal recessive neuromuscular disease, caused by the homozygous loss of function of the *survival motor neuron* (*SMN*) 1 gene (1). Onset of SMA is before the age of 3 years in the large majority of patients (2).

Age at onset in childhood and achieved motor milestones are used to classify SMA into type 1 (onset in the first 6 months of life, non-sitters), type 2 (onset between 6 and 18 months, sitters) and type 3 (onset between 18 and 36 months (type 3a) or after 36 months but before 18 years (type 3b), walkers).

The introduction in the past few years of disease-modifying treatments (DMTs) has changed the prospects of children with SMA considerably. A large majority now experiences motor function improvement or stable disease (3) instead of the steady decline that defines its natural history. Improved outcome may imply new phenotypes and disease trajectories that require different approaches in supportive care, such as treatment of joint contractures (4, 5). The 2017 international standards of care (6) provide important pro-active guidance for contracture management in SMA, but recommendations are based on experiences in a population of individuals with SMA who were not treated with DMTs.

In the Netherlands, reimbursement for treatment with DMTs has become available incrementally since May 2017. Newborn screening was introduced in June 2022. Given the motor milestone improvement seen with the use of DMT (3), there was a need to reconsider contracture management in individuals with SMA. Prior to the introduction of DMTs, contracture management was an important part of rehabilitation therapy; however, a poor clinical prognosis did not appear to motivate individuals with SMA (7), their caregivers, or healthcare professionals in the Netherlands to adhere to a strict contracture prevention regimen. Considerations for contracture management vary significantly per individual as they depend on factors such as age, disease progression, joint range of motion, and functional level. In the Netherlands, many pediatric rehabilitation physicians report a lack of clarity in contracture management with the introduction of DMTs. The question raised among Dutch pediatric rehabilitation physicians is, therefore: ‘*how should one apply contracture management in a changing landscape in children with SMA?*’

This study aimed to describe current approaches to contracture management among Dutch pediatric rehabilitation physicians caring for children with SMA receiving DMT, and to explore the underlying considerations and clinical reasoning that inform their decisions on contracture management in the evolving therapeutic landscape of SMA.

## 2 Materials and methods

We performed a two-part sequential mixed-methods study, comprising (1) a cross-sectional survey among Dutch pediatric rehabilitation physicians involved in contracture management of children with SMA, and (2) an advisory group meeting with (pediatric) rehabilitation physicians, designed to reflect on their care practices and their considerations in preventive and therapeutic contracture management. This study was not subject to Dutch Medical Research Involving Human Subjects Act. All respondents gave informed consent in the (online) survey. Respondents could not proceed before completing the consent form.

### 2.1 Survey

We developed the survey questions through discussions among members of the study team and modified them in response to the feedback from multidisciplinary team members (researchers, rehabilitation physicians and physical therapists) of the Netherlands SMA Center at the University Medical Center Utrecht (UMCU). Two rehabilitation physicians piloted the survey, which consisted of 24 questions aimed at identifying pediatric rehabilitation physicians’ views and practice regarding contracture management in SMA. The survey contained both open-ended questions (i.e., asking respondents to answer in their own words) and closed-ended questions (multiple-choice items, partly supplemented with an optional open-text field for additional comments) and took approximately 20 min to complete.

#### 2.1.1 Context of treatment availability in the Netherlands during the study period

Nusinersen first became available in May 2017 for SMA type 1 and from January 2018 for young children (up to the age of 6 years) with SMA types 2 and 3 (compassionate use and early access programs, respectively) and was reimbursed for children up to the age of 9.5 years at the start of treatment as of August 2018. Reimbursement of gene therapy (Zolgensma®) was implemented in December 2021 for presymptomatic cases with 2 or 3 SMN2 copies. At the time of the survey nusinersen (Spinraza®) in December 2021 for presymptomatic cases with 2 or 3 SMN2 copies. At the time of this study nusinersen was the most administered DMT in the Netherlands. Newborn screening was introduced a few weeks before the advisory group meeting.

We identified three standardized case scenarios representing groups of children with varying disease severity:

1. Presymptomatically treated group: children who started with DMT prior to the onset of clinically manifest symptoms.
2. Symptomatically treated, group 1: symptomatic children who initiated DMT within 2 years after diagnosis.
3. Symptomatically treated, group 2: symptomatic children who started with DMT more than 2 years after diagnosis.

Questions for this survey are based on both the Dutch guideline SMA type 1 (8), as well as the International Standards of Care for SMA (Diagnosis and Management of SMA: Part 1 Recommendations for diagnosis, rehabilitation, orthopedic and nutritional care) (6).

Because the registry of Dutch pediatric rehabilitation physicians does not include data on diagnosis groups (i.e., whether a physician works with children with SMA), we invited all pediatric rehabilitation physicians (n = 151) to the survey. We first asked the physicians whether they worked with children with SMA. If the rehabilitation physician was not treating a child or children with SMA, the survey ended immediately. Data collection took place using Castor’s Electronic Data Capture software.^1^ We sent a reminder to the non-responders after 2 weeks.

The survey consisted of 4 parts (survey included as Supplementary file 1):

#### 2.1.2 Part 1: general questions

In this section, questions are asked about work setting—(academic) hospital or rehabilitation center—and number of children with SMA in care.

#### 2.1.3 Part 2: questions on measuring joint range of motion

In this section, questions about measurements of joint range of motion are asked.

#### 2.1.4 Part 3: contracture management, indication and purpose

In this section, the questions are based on international standards of care (6) (i.e., pain and decrease in function) and expert opinion. Respondents rated the extent to which they agreed with the recommendations of a certain treatment goal, by answering on a 5-point Likert scale: totally agree, agree, neutral, disagree or totally disagree. The full list of survey questions is provided in the Supplementary file 1.

#### 2.1.5 Part 4: questions about contracture management in relation to guidelines

This section of the questionnaire aimed to evaluate the extent to which the current clinical decision-making in the era of DMT aligns with the Dutch national guideline SMA (8) and the international Standards of Care (6). To this end, respondents were presented with descriptions of the 3 treatment groups. Respondents rated the extent to which they agreed with each recommendation on a 4-point Likert scale: I strongly disagree with the statement, disagree, agree or strongly agree.

Then, the respondents ranked each recommendation for the three different treatment groups: presymptomatically treated group, symptomatically treated group 1 & 2. The full list of survey questions is provided in the Supplementary file 1.

### 2.2 Advisory group meeting

In the survey, respondents could consent to being approached for a later in-depth interview about the survey results. For the advisory group meeting, we invited pediatric rehabilitation physicians who participated in the survey and consented to being approached for the in-depth questions. Selective sampling optimized the representation of the Dutch pediatric rehabilitation physicians population. We invited a total of 14 physicians for the meeting, aiming for a broad variation, consisting of physicians working in different work settings (both hospital and rehabilitation center). Physicians in a University hospital typically carry out check-ups on a large group of children with SMA once or twice per year; physicians working in rehabilitation centers schedule appointments with children with SMA on a more regular basis. In addition to the pediatric rehabilitation physicians working with (young) children, a rehabilitation physician working with (young) adults was asked to join the advisory group meeting, in order to add a different point of view. We invited this physician to include experiences with adults regarding contractures and (previous) contracture management.

In the advisory group meeting, we discussed views on contracture management with the aim of obtaining insight into the underlying considerations and clinical reasoning that inform their decisions on contracture management in the evolving therapeutic landscape of SMA. We preferred the interaction of a group meeting over survey questions or (individual) interviews, in order to facilitate a dialogue among rehabilitation physicians and to create a consensual joint vision for future directions. The central theme of the advisory group meeting was to discuss dilemmas and considerations in the clinical decision-making process regarding (preventive) treatment of contractures in children with SMA. The topic guide was developed based on survey outcome of parts 3 and 4.

The moderator invited participants to share their considerations and experiences regarding contracture treatment through broad exploratory questions, followed by more specific questions that instigated discussion. The session ended with questions on how we should design future contracture treatment within the changing landscape for SMA, particularly after the introduction of DMTs and of newborn screening (topic guide is included as Supplementary file 2).

The 90-min session was conducted online via Microsoft Teams (an online communications and collaboration platform). One of the authors (AB) with experience and expertise in focus group facilitation moderated the session. At the beginning, participants were briefed on the purpose and rules, including: introduction of all participants; explanation of the use of recordings; respecting participant privacy and opinions; confidentiality of participant identity. The moderator ensured all participants had an opportunity to express their thoughts. We recorded the session and analyzed the transcript to derive various themes. We then carried out a member check and asked participants to check the key messages and complete them with five personal recommendations regarding contracture treatment.

### 2.3 Data collection

We collected survey data in July 2021. The online survey started on July 1st, followed by a reminder on July 19th. The advisory group meeting was held 1 year later on July 8th 2022.

### 2.4 Data analysis

When ≥75% of the survey had been completed per respondent, data were analyzed with descriptive statistics using IBM SPSS Statistical data software (IBM SPSS Statistics for Windows, Version 26.0. Armonk, NY).

Three of the authors (IOL, AB, JWG) further analyzed the transcript to identify important clinical themes.

## 3 Results

### 3.1 Results from the survey

#### 3.1.1 Parts 1 and 2: general questions

A total of 85 of 151 invited physicians responded to the survey (response rate 56%), 41 of whom indicated that they were not involved in the care of children with SMA. Six respondents did not complete the online survey (completion of survey <75%), leaving 38 surveys for analysis. Of these 38 respondents, 9 work in a university hospital (24%), 8 in a general hospital (21%), 26 in a rehabilitation center (68%) and one works in another unspecified setting. Six respondents work in two settings.

The annual patient load of 29 respondents (76%) was 1–5 children with SMA, of 5 (13%) it was 5–10 children, of three rehabilitation physicians (8%) it was 10–20 children and one (3%) looked after more than 20 children with SMA.

#### 3.1.2 Part 3: contracture management, indication and purpose

The importance of contracture management was given a median rating of 8 out of 10 [min-max 7–10]. Responses to open-ended question 3.2 indicated that the increased importance of contracture management following the introduction of disease-modifying therapies (DMTs) is primarily attributed to improved functional prognosis, increased life expectancy in SMA type 1 and the significant impact of contractures on limiting further motor development, even when muscle strength improves. In contrast, rehabilitation physicians who perceive no change in the importance of contracture management emphasize that contracture treatment was already a major focus prior to DMT. They describe similar indications for intervention, but note that the perceived effects of these interventions have changed and are now considered to differ.

The perceived importance of specific treatment goals, derived from the closed-ended questions, is presented in Table 1. Maintaining function, sitting posture, and preventing pain were rated as the most important objectives by respondents.

**Table 1:** Purpose of contracture management in children with SMA according to pediatric rehabilitation physicians (part 3).

Purpose	Strongly agree n = (%)	Agree *n* = (%)	Neutral *n* = (%)	Disagree *n* = (%)	Strongly disagree *n* = (%)
To maintain function	**12 (31.6%)**	**21 (55.3%)**	4 (10.5%)	0 (0%)	1 (2.6%)
To prevent pain	**8 (21.1%)**	**28 (73.7%)**	0 (0%)	2 (5.3%)	0 (0%)
Optimal sitting posture	**15 (39.5%)**	**23 (60.5%)**	0 (0%)	0 (0%)	0 (0%)
Patient appearance	2 (5.3%)	**19 (50%)**	**13 (34.2)**	3 (7.9%)	1 (2.6%)
At parents request	0 (0%)	6 (15.8%)	**18 (47.4%)**	**9 (23.7%)**	5 (13.2%)
Avoiding the need for surgery	2 (5.3%)	**25 (65.8%)**	**7 (18.4%)**	3 (7.9%)	1 (2.6%)

^*^Highest and second highest percentages are presented in bold.

Concerning the timing of standing frame introduction (part 3.5), one participant initiated use between 0 and 1 year of age, 23 between 1 and 2 years, 13 between 2 and 5 years, and one after the age of 5 years. Reasons for discontinuation of contracture management reported in the open-ended responses (part 3.6) include:

- The intervention was considered disproportionately burdensome relative to the anticipated benefits for the child and family;
- The degree of contracture had progressed to a point where the use of a standing frame or orthoses no longer yielded clinically meaningful outcomes;
- Motivation on the part of the child and/or parents was insufficient to justify continuation of the intervention;
- The child experienced pain associated with the use of the standing frame or orthoses;
- Ongoing contracture management was deemed unlikely to result in additional functional gains.

Discontinuation was initiated at the request of the child or family.

#### 3.1.3 Part 4: contracture management, prevention/treatment in relation to guidelines

Tables 2 and 3 lists the topics for which Dutch pediatric rehabilitation physicians are less likely to follow the advice/recommendations of the guidelines [ratings, by Dutch pediatric rehabilitation physicians, of statements derived from guidelines (part 4)]. Regarding the recommendations of the Dutch guideline for SMA type 1 (Table 2), the main results are: 37–48% (strongly) disagreed with the statement that muscles should be stretched at least 3-5x per week. One-third of the respondents disagreed with using static and/or dynamic orthosis in the presymptomatically treated group. All but 1 respondent (strongly) agreed with using orthosis in the two symptomatically treated groups. Thirty to 36 %, depending on the subgroup, (strongly) disagreed with the statement: use a brace if postural scoliosis is present.

**Table 2:** Ratings of various statements, derived from the Dutch guidelines on SMA type 1, by Dutch Pediatric Rehabilitation Physicians (Dutch guideline SMA type 1: Published online 2018. Startpagina - Spinale musculaire atrofie (SMA) type 1 - Richtlijn - Richtlijnendatabase).

Statement/advice		Presymptomatically treated group (subgroup 1)		Sypmptomatically treated, group 1 (subgroup 2)		Symptomatically treated, group 2 (subgroup 3)	
Level of agreement with the statement, *n* (%)		(Strongly) agree	(Strongly) disagree	(Strongly) agree	(Strongly) disagree	(Strongly) agree	(Strongly) disagree
Stretch muscles and maintain their length, within the pain threshold, at least three to five times a week, sustaining the final position for at least 30 s.	I advise parents to stretch muscles in accordance with the previous statement.	14 (52%)	**13 (48%)**	17 (63%)	**10 (37%)**	17 (63%)	**10 (37%)**
Combat contractures by performing prolonged stretching. Static and dynamic orthoses can be used for this purpose.	I use static and/or dynamic orthosis in this patient group.	17 (63%)	**10 (37%)**	26 (96%)	**1 (4%)**	27 (100%)	**0 (0%)**
Consider using a brace for children with SMA type 1C or children receiving drug therapy if postural scoliosis is present.	I consider the use of a brace for children with SMA type 1C or children treated with DMTs, if postural scoliosis is present.	18 (64%)	**10 (36%)**	19 (70%)	**8 (30%)**	18 (66%)	**9 (34%)**

We asked participants to rank different pieces of advice, indicating the extent of their agreement with that recommendation. Ranking options were: I strongly agree, agree, disagree or strongly disagree with this advice. The participants ranked each statement for the following (sub)groups: (1) Presymptomatically treated group: children who started with DMT prior to the onset of clinically manifest symptoms. (2) Symptomatically treated, group 1: children who initiated DMT within 2 years after diagnosis. (3) Symptomatically treated, group 2: children who started with DMT more than 2 years after diagnosis. ^*^Percentages (strongly) disagree per subgroup are presented in bold.

**Table 3:** Ratings of various statements, derived from the International Standards of Care for SMA, by Dutch Pediatric Rehabilitation Physicians; Diagnosis and management of spinal muscular atrophy: Part 1: Recommendations for diagnosis, rehabilitation, orthopedic and nutritional care by Mercuri et al. (6).

Statement/advice		Presymptomatically treated group (subgroup 1)		Symptomatically treated, group 1 (subgroup 2)		Symptomatically treated, group 2 (subgroup 3)	
Level of agreement with the statement, *n* (%)		(Strongly) agree	(Strongly) disagree	(Strongly) agree	(Strongly) disagree	(Strongly) agree	(Strongly) disagree
Non-sitters	For children who cannot sit independently, I recommend a minimum stretching frequency of 3–5 times a week.	17 (63%)	**10 (37%)**	21 (78%)	**6 (22%)**	21 (78%)	**6 (22%)**
	For children who cannot sit independently, I recommend daily use of hand splints for range of motion and functioning.	9 (37%)	**17 (63%)**	13 (50%)	**13 (50%)**	14 (54%)	**12 (46%)**
	For children who cannot sit independently, I recommend the daily use of AFOs and/or KAFOs for range of motion.	17 (65%)	**11 (35%)**	23 (85%)	**4 (15%)**	23 (85%)	**4 (15%)**
	For children who cannot sit independently, I recommend daily use of TLSO for positioning.	16 (62%)	**10 (38%)**	18 (67%)	**9 (33%)**	18 (67%)	**9 (33%)**
Sitters	For children who can sit independently, I recommend a minimum frequency of stretching of 5–7 times a week.	17 (63%)	**10 (37%)**	20 (74%)	**7 (26%)**	20 (74%)	**7 (26%)**
	For children who can sit independently, I recommend using a standing table for a maximum of 60 min, with a minimum frequency of 3-5× per week and an optimal frequency of 5-7× per week.	21 (78%)	**6 (22%)**	23 (93%)	**2 (7%)**	23 (93%)	**2 (7%)**
Ambulant	For ambulatory children, I recommend active stretching 2-3× per week (with an optimal frequency of 3-5× per week).	16 (59%)	**11 (41%)**	22 (81%)	**5 (19%)**	23 (85%)	**4 (15%)**

We asked participants to rank different pieces of advice, indicating the extent of their agreement with that recommendation. Ranking options were: I strongly agree, agree, disagree or strongly disagree with this advice. The participants ranked each statement for the following (sub)groups: (1) Presymptomatically treated group: children who started with DMT prior to the onset of clinically manifest symptoms. (2) Symptomatically treated, group 1: children who initiated DMT within 2 years after diagnosis. (3) Symptomatically treated, group 2: children who started with DMT more than 2 years after diagnosis. ^*^Percentages (strongly) disagree per subgroup are presented in bold.

Results on the level of agreement about statements by the international SoC for SMA (Table 3). For non-sitters, 46–63% of the respondents (strongly) disagreed with the statement ‘*I recommend daily use of hand splints for range of motion and functioning*’ in all three subgroups. For subgroups 2 and 3, only 15% disagreed with the statement *‘For children who cannot sit independently, I recommend the daily use of AFOs and/or KAFOs for range of motion*’. In sitters, 26–37% of the respondents (strongly) disagreed with the statement *‘I recommend a minimum frequency of stretching of 5–7 times a week’* in all three subgroups.

In the ambulant group, 41% (strongly) disagreed with the statement ‘*I recommend active stretching 2-3x per week*’ in the presymptomatically treated group.

### 3.2 Dutch expert advice and recommendations

A total of six rehabilitation physicians, consisting of five pediatric and one adult rehabilitation physician, were able to join the advisory group meeting. Three of the pediatric rehabilitation physicians work in a rehabilitation center; the other two and the adult rehabilitation physician work in a university hospital. All rehabilitation physicians involved in this study have a dedicated focus on neuromuscular disorders in their clinic, have a minimum of 5 years of experience of treating children with SMA (three of them had over 10 years of experience) and they are all members of the national working group on neuromuscular disorders (additional information on advisory group experts is included as Supplementary file 3). We asked the participants of the advisory group to indicate their caseload (at the time of participation) of children with SMA, which varied from a minimum of one child to over 30 children.

Three main themes emerged from the analysis of the advisory group deliberations: (1) functional prognosis as key element in decision-making; (2) clinical uncertainty of contracture interventions, and (3) considerations in the light of ICF-based functioning: incorporating contextual factors.

#### 3.2.1 Functional prognosis as key element in decision-making

All advisory group experts agreed about functional prognosis being a key element in decision-making by pediatric rehabilitation physicians regarding contracture treatment. Participants stressed the importance of the following factors to be taken into account for a functional prognosis: number of SMN copies, age, age at start of treatment with DMT, personal and environmental contextual factors, muscle strength and range of motion.

Participant: ‘I notice that in a non-ambulatory patient, regarding contracture management of the legs, I am more likely to go along with parents when they indicate discontinuing contracture treatment. When hands are involved, I am more keen on continuing contracture treatment to see if we can come up with other tricks or ways to continue contracture treatment.’

Participants stressed the importance of regular and specific physical examinations (i.e., range of motion, muscle strength) and monitoring side-effects of contracture management and combining this information with the expected functional prognosis of the child to evaluate the effects of contracture treatment.

Participant: ‘A brother and sister, both SMA type 3, have a completely different foot position. So functional prognosis is not only based on SMN copy number.’

With frequent monitoring of physical function and treatment effects, a pediatric rehabilitation physician has the opportunity to initiate or switch contracture intervention in consultation with child and/or parent.

#### 3.2.2 Clinical uncertainty of contracture interventions

The advisory group experts agreed on the difficulty of predicting the outcome of contracture treatment for an individual child. The uncertainty of the benefit for the individual child in the immediate and more distant/longer-term future was mentioned as being a problem; therefore, the group agreed that it was important to communicate this uncertainty to parents.

The group discussed the lack of evidence on the use of orthoses, specifically on the use of Knee-Ankle-Foot-Orthoses. There was no consensus on the effectiveness of KAFOs. There are unanswered questions about KAFOs, namely (1) what type of orthosis should be advised (static, dynamic, type of hinge that should be used)? (2) should KAFOs be used in young children, taking into consideration the tolerability and potential negative side-effects (for example, pressure ulcers or sleep deprivation due to wearing splints at night)?

#### 3.2.3 Considerations in the light of ICF-based functioning: incorporating contextual factors

The advisory group reached consensus about the use of the International Classification of Functioning, Disability and Health (ICF) framework (9) as a helpful tool in clinical decision-making and in creating a person-centered care plan. This ICF framework (9) is a tool, commonly used in Dutch rehabilitation care, to describe an individual’s level of functioning, that includes ‘body function/structure’, ‘activities/participation’ and contextual factors (personal and environmental factors) (9). The ICF describes an individual’s level of functioning in terms of ‘body function/structure’, ‘activities/participation’ and contextual factors (personal and environmental factors) and facilitates goal-setting and evaluation of effects of contracture management. The group indicated the importance of routine evaluation of the following factors over time: sleep, the social–emotional and cognitive development of a child, living situation of a child and family circumstances.

##### 3.2.3.1 Examples/quotes by participants

‘Sometimes a living situation does not allow a child to stand in a stander at home.’

‘The parenting skills could influence the decision-making.’

Also, participants of the advisory group meeting described a feeling amongst parents of being willing to try anything for their child, and feelings of guilt, doubt and uncertainty in relation to doing everything for the wellbeing of their child, as part of being a ‘good parent’. Examples of why parents experience these feelings:

1. Feelings of guilt/doubt when they stop a certain type of contracture treatment;
2. Feelings of guilt/doubt when they want to continue the treatment, but the orthosis/family factors/child/situation makes it impossible for them to continue with it;
3. Feelings of guilt/doubt when parents continue the treatment, despite the contracture worsening, and even though they do their utmost, the contractures worsen over time.

After the member check and the final input of participants’ most important recommendations, we collated the recommendations of all participants.

##### 3.2.3.2 Functional prognosis

- Address physical activity and prevention of secondary impairments and health conditions in the care plan.
- Promote active range of motion to maintain muscle length (if necessary with the help of devices like arm support).
- Determine the main focus in non-ambulant children with SMA; functional prognosis of the arms and hands could be of great importance and are usually the main focus in non-ambulant children.

##### 3.2.3.3 Clinical uncertainty of contracture intervention

- Address the uncertainty, not only because of lack of scientific evidence but also practical and personal uncertainties (10) (what works for this individual child) and be honest about it.
- Even though there is a lack of evidence, in individual cases, the advice is to try different interventions to prevent and/or delay contractures.

##### 3.2.3.4 Incorporating contextual factors

- Take individual factors into account: for example, is it feasible for the child, how does the child sleep and how does the child feel emotionally?
- Take environmental factors into account: is it feasible for the family/parents and the siblings?
- Support children and families in the choices they make and address social–emotional goals in the care plan.
- Also, the advisory group experts explicitly recommend to ask adolescents whether they think the contractures affect their appearance and how this impacts them. This could be a potential goal of contracture treatment.
- Communicate with parents and/or a child: what experience do you want to give a child with regard to standing/walking during his childhood?

## 4 Discussion

Dutch pediatric rehabilitation physicians experience challenges in clinical decision-making regarding contracture management in the absence of clear clinical guidance in a rapidly evolving therapeutic landscape for SMA. They urge the importance of ‘optimizing sitting posture’, ‘prevention of pain’ and of ‘maintaining function’ as treatment goals in contracture management of children with SMA. Furthermore, advisory group experts highlighted that careful consideration of the broader ICF perspective on functioning is essential when jointly developing a contracture management plan with the child with SMA and their parents. They emphasized that, beyond body functions and structures, such a plan should also take into account personal and environmental factors that influence the child’s activity and participation.

Finally, advisory group experts reported difficulties in giving clear recommendations on the necessity of orthoses and/or passive stretch management, as evidence to support these interventions is lacking.

Historically, treatment-naïve children with SMA developed progressive weakness with secondary contractures (4, 5, 11). Due to lack of evidence-based interventions (12–15) contracture management in SMA has been highly challenging and has complicated rehabilitation recommendations more broadly. Therefore, the international standards of care (SoC) were carefully developed based on expert opinions from numerous international specialists. However, these recommendations were derived from experiences in patient populations who had not been treated with DMTs (6). With the introduction of DMTs, the clinical phenotype of SMA has changed considerably. Over the past years, rehabilitation physicians have had to redefine their perspectives on contracture management, not only in response to the improved prognosis and evolving functional abilities of these children, but also owing to the limited empirical evidence available to guide clinical decision-making. Although rehabilitation physicians are familiar with the SoC, difficulties were experienced in implementing recommendations of the SoC in the personalized care of children treated with DMTs, in this highly heterogenous group.

Respondents described the importance of ‘prevention of pain’ and ‘avoiding the need for surgery’ as targets for contracture management. However, the risk of pain and future surgery in SMA is unclear. Lager and Kroksmark showed that (16) showed that pain is a frequent problem amongst adolescents (12–18 years old) with SMA in Sweden, however no specific information was provided on pain due to contractures. Incidence of pain in adults with NMD varies amongst studies from 40 to 75% (17–19). It is unlikely that soft tissue contractures are, in themselves, a source of pain at rest (19). Pain seems to become apparent when joints are pushed to the end of their range. It would be of benefit to have a better understanding of the impact of contractures amongst adults, i.e., pain, past surgery, problems with sitting posture due to contractures, problems with fitting shoes. Approximately half of the respondents (47.4%) rated the goal “at the request of parents” as neutral, highlighting the need to clarify its meaning. Understanding why parents request contracture treatment and supporting them through in-depth discussions is essential. Clinicians should collaboratively set, monitor, and revisit treatment goals with parents and children, regularly weighing potential benefits and adverse effects as part of shared decision-making in contracture management for children with SMA, and engage in open discussions with parents regarding uncertainties arising from limited clinical experience and the evolving understanding of disease trajectories in the early post-DMT era. This can help inform other on how to communicate when new treatment options become available for other neuromuscular diseases.

We observed noteworthy results on the contracture management in the group of presymptomatic treated children. Across all categories (non-sitters, sitters, and ambulant), higher proportions of respondents expressed (strong) disagreement with the statements of the SoC compared to the symptomatically treated groups. The survey did not provide data explaining why respondents were less likely to recommend different contracture management strategies for this group. For the ambulant group, one possible explanation is that respondents assumed these children would not develop contractures, or would do so to a lesser extent, compared to those in the symptomatically treated groups. These unexpected findings may also reflect the clinical challenges faced by pediatric rehabilitation physicians in this emerging field, as they were asked to consider children with whom they had no prior clinical experience. Mortenson et al. (20) recently highlighted that empirical evidence is needed to understand the best rehabilitation approaches for SMA post DMTs, in response to the uncertainties Canadian therapists experienced in the care for children with SMA receiving DMT.

The advisory group experts stressed the importance of a careful individual evaluation of which intervention would be of help while taking into account personal and environmental factors. Therefore, we emphasize the importance of using the International Classification of Functioning, Disability, and Health (ICF), as it provides a comprehensive and multidimensional framework allowing to update rehabilitation models and individualized care plans encompassing body functions and structures, activities and participation, as well as personal and environmental factors (9). The ICF framework helps contextualize contracture management by linking impairments in body function and structure to broader functional goals, enabling personalized, holistic rehabilitation planning. The ICF also accounts for contextual factors such as home setup, caregiver support, and motivation. These can significantly influence the success of contracture interventions and should be considered when designing rehabilitation plans.

An illustrative example from this study highlights the use of the ICF framework: two children with similar disease progression may follow different contracture plans due to personal and environmental factors. For instance, a child with communication difficulties may derive greater long-term benefit from investing his/her (limited) energy in speech and language therapy rather than in interventions targeting hip and knee contractures. This underscores the importance of tailoring individualized contracture treatment goals to each child’s unique context, priorities, and potential functional gains in the ICF framework. Another illustrative example, in relation to the use of the ICF framework and focusing on personal and environmental factors, concerns the feelings of guilt and doubt described by parents in relation to doing everything for the wellbeing of their child, Gibson et al. (21) referred to similar experiences of parents with rehabilitation treatment. In a qualitative study amongst parents and children with cerebral palsy (CP), they described beliefs about the value of walking held by children with CP and their parents, and how these beliefs inform rehabilitation choices and perceptions of ‘success’. They described that tapering of walking interventions contributed to feelings of guilt and doubt. Also there might be feelings of disappointment for a health care professional. They might have a desired outcome, which does not fit with progression of contractures. This desired outcome might have important implications for the individual choices of a health care professional. Whereas one individual might see a straight leg as a desired outcome and is prepared to try everything to achieve the goal, another might see the preferred goal as secondary and be much less likely to try multiple options (22).

The strengths of this study include the number of rehabilitation physicians that responded to the survey, which lends robustness and representativeness to the survey. This study was conducted within the first few years after the introduction of DMTs in the Netherlands, a period during which the functional prognosis of individual children receiving DMTs was still uncertain. Although some information was available from clinical trials, this study represents the first real-world clinical experience gained by Dutch rehabilitation physicians themselves. In the absence of empirical evidence, it is essential to share clinical experiences internationally to bridge knowledge gaps.

A potential limitation is that the survey data did not assess reasons for non-adherence across the entire sample; this aspect was explored only in the in-depth qualitative part of the study. This study is limited with regard to the reflection of presymptomatic children. When designing the survey, we aimed to explore whether physicians would develop different treatment plans for children in the presymptomatic group who started DMT before the onset of symptoms, compared to children with SMA who started their treatment later in life, often with pre-existing contractures. At that time we had little knowledge of what DMTs would do for newborns (both symptomatic and presymptomatic). Upon discussing the survey results, we noted that pediatric rehabilitation physicians found these questions challenging to answer, due to the limited clinical experience available with the presymptomatically treated group at the time of the survey. Some questions were too hypothetical. In the new generation of children with SMA, it is important to regularly assess joint mobility and monitor the development of contractures over time, and to systematically record these data in a registry, enabling longitudinal analyses to better understand the prognosis of contractures and the factors that influence it. In the Netherlands, there is frequent monitoring of joint range of motion, both by the pediatric rehabilitation physician and the involved physiotherapist(s). This is important in the light of the difficulties experienced in the timing of initiation of contracture treatment. Pediatric rehabilitation physicians may be uncertain when to start the treatment in asymptomatic children with SMA. It is a very promising group in terms of their functional prognosis, which should, therefore, motivate clinicians to start early in order to prevent contractures. There is, however, a trade-off, as children might only develop minor contractures later on in life and are potentially being treated unnecessarily during childhood. A recent qualitative study (7) amongst adolescents and young adults with SMA showed that there is little motivation for standers/orthosis in general, but when there is a clear treatment goal, motivation increases. Also, recent initiatives in rehabilitation care within the evolving therapeutic landscape (20), including specific attention to contracture management (23), represent promising developments for this new generation of children.

Finaly, despite the small sample of pediatric rehabilitation physicians involved in the care for children with SMA, we are convinced that our study included a representative sample from diverse work settings, encompassing a sufficient range of experience with SMA. Our results may not, however, be generalizable internationally.

## 5 Conclusion

In line with Trabacca et al. (24) we stress the importance of using the ICF framework when updating practice guidelines/the standards of care regarding contracture management, for people with SMA in this era of disease-modifying therapies. The Dutch approach focuses on multidisciplinary care, collaboration between healthcare professionals and a patient-centered approach, with a focus on activities and participation, rather than solely on body functions and structures, taking into account personal and external factors. The use of the ICF framework is essential for developing better rehabilitation practice guidelines, specifically for individual contracture guidance. With an optimized individual contracture plan, defining child-specific treatment goals which work towards optimizing the level of participation, the quality of life can be improved for children with SMA.

## Data Availability

The raw data supporting the conclusions of this article will be made available by the authors, without undue reservation.

## Footnotes

1
www.castoredc.com
