# Supplementary material for: Dutch rehabilitation physicians’ perspectives on contracture management in children with spinal muscular atrophy: challenges in a changing landscape
Source: Front Neurol. 2025 Dec 10;16:1670391. doi: 10.3389/fneur.2025.1670391 (PMC12727611; doi:10.3389/fneur.2025.1670391)
Supplement: Supplementary file 1 [file Table_1.docx]

|  | Years of experience in treating children with SMA | Dedicated focus on NMD in clinic? | Member of national working group on NMD? | Work setting? | Caseload of children with SMA at the time of participation? |
| --- | --- | --- | --- | --- | --- |
| Expert 1 | >10 years | Yes | Yes | Rehabilitation center | 1-5 |
| Expert 2 | 5-10 years | Yes | Yes | Rehabilitation center | 5-10 |
| Expert 3 | >10 years | Yes | Yes | Rehabilitation center | 5-10 |
| Expert 4 | 5-10 years | Yes | Yes | University hospital | 10-20 |
| Expert 5 | 5-10 years | Yes | Yes | University hospital | >30 |
| Expert 6 | >10 years | Yes | Yes | University hospital | >30 |

**Additional information advisory group experts**
