# Supplementary material for: Dutch rehabilitation physicians’ perspectives on contracture management in children with spinal muscular atrophy: challenges in a changing landscape
Source: Front Neurol. 2025 Dec 10;16:1670391. doi: 10.3389/fneur.2025.1670391 (PMC12727611; doi:10.3389/fneur.2025.1670391)
Supplement: Supplementary file 2 [file Data_Sheet_1.pdf]

Topic guide for discussion at the advisory group meeting

Thoughts on (preventive) contracture management in children with SMA

Important:

- All questions are posed in the context of SMA (types 1-3, age range 0-18 years old).
- We want to understand why Dutch rehabilitation physicians make specific choices and why these vary between them.
- There is no right or wrong, all information is useful.
- We made an audio recording.

Goal-setting:

1. Questioning the general principles
2. Prompting

General principles:

1. What comes to mind about contracture treatment in the context of a child with SMA?
2. In your opinion: which is the most problematic contracture and why?
3. Which factors do you take into account, and what do you consider, when making a treatment plan for contractures?
4. Do you sometimes argue with a physical therapist/occupational therapist/psychologist or other health care professional about contracture management of a child? Which factors in the discussion are the most important?
5. What is your opinion about the impact of contracture management? Do you feel your experience plays a role in decision-making?
6. In your opinion, what would be the most important improvement in care with regard to contracture management? Or, what do you think is missing from the contracture management protocol in the Netherlands?
7. Are there views on other conditions that you take into account in your decision-making?

Prompting:

1. What is the most commonly referred to 'contracture of hinderance' in adulthood? What are the most important questions about contractures in (young) adults?
2. What is your opinion about contracture management in the 'new group' of children with newborn screening, presymptomatic- or early symptomatic-treated children?
3. What is your opinion about preventive contracture management in relation to symptomatic contracture management?
