# Supplementary material for: Dutch rehabilitation physicians’ perspectives on contracture management in children with spinal muscular atrophy: challenges in a changing landscape
Source: Front Neurol. 2025 Dec 10;16:1670391. doi: 10.3389/fneur.2025.1670391 (PMC12727611; doi:10.3389/fneur.2025.1670391)
Supplement: Supplementary file 3 [file Data_Sheet_2.docx]

**Survey for pediatric rehabilitation physicians involved in the care of children with SMA: contracture treatment.**

**The e-mail below, addressed to pediatric rehabilitation physicians, concerns the care of children with a neuromuscular disorder.**

Dear colleague,

The care for children with SMA has changed considerably in recent years due to the introduction of new drugs for SMA, nusinersen (Spinraza®) currently being the most important. Due to the effects of these treatments on the motor development of a large number of these children, contracture management can now be seen in a different light. In this project, we want to learn together how we can best shape contracture treatment in children with SMA. It is important to first describe the initial situation and to identify current bottlenecks in the (preventive) treatment of contractures. Our ultimate goal is to optimize the care for children with SMA.

We would ask you to complete the online questionnaire regarding the current contracture policy. The questions/statements are based, among other things, on the international "Standards of care" for SMA and the Dutch guideline for SMA type 1. The results of these surveys have been included in a broader study into the impact of contractures on daily functioning. Completing the questionnaire will take you about 20-30 minutes. Even if you are not involved in the care of children with SMA, would you please open the digital questionnaire and answer the first 2 questions. If you indicate in question 2 that you are not treating children with SMA, the questionnaire will automatically stop.

**Part 1: General questions**

1. You work as a rehabilitation doctor in a (multiple options possible):

*university hospital, (community) hospital, rehabilitation center, other...*

1. How many children with SMA do you see, or are receiving treatment by you, per year, on average?

*0, 1-5, 5-10, 10-20, >20 (If you answer 0, the questionnaire will stop)*

**Part 2: Measurement of contractures**

1. How many times a year do you see a child with SMA for a check-up (every contact moment, incl. technical/shoe consultation)?

*Occasionally, 1x per year, 2x per year, 3-5x per year, >5x per year*

1. How do you monitor joint mobility (range of motion) in children with SMA? (multiple options possible)

*Measurement by rehabilitation physician, measured by team member (physical/occupational therapist), measured by (physiotherapist/occupational) therapist in the community, this is not measured.*

1. Which joints are measured?

*Shoulder/elbow/wrist/fingers/hip/knee/ankle/other, namely....*

1. How often do measurements take place in children <5 years of age with SMA type 1 or 2?

*Very frequently (quarterly or more often), regularly (about every six months), occasionally (approximately annually)*

1. *Open text field for explanation* How does documentation of joint mobility take place in your center (multiple answers possible)?
   1. *As text in electronic patient dossier (EPD) / e-chart*
   2. *In a numeric field in EPD*
   3. *Graphics EPD*
   4. *Separate database outside the EPD*
   5. *Other, i.e.*

**Part 3:** **Contracture management, indication and purpose**

Thoughts on the importance of contracture treatment

1. Please rank the importance of contracture prevention (rating from 0 (lowest) to 10 (highest)).

*Open text field for additional comments (optional)*

'An important number of the children with SMA in the Netherlands is currently being treated with DMT, nusinersen (spinraza®) being the most important. Due to the effects of these treatments on the motor development of a large proportion of these children, the contracture management policy has also been placed in a different light.'

1. Do you think the need for contracture prevention has changed since the implementation of DMT (in this case nusinersen (spinraza®))?

*Yes/no*

*(Mandatory open-text field at yes): The need for contracture prevention has changed, because..*

*(Mandatory open-text field at no): The need for contracture prevention has not changed, because...*

1. Please indicate the extent to which you agree with the following statements. 0=n/a, 1=strongly agree, 2=agree, 3=neutral, 4=disagree, 5=strongly disagree
   - Contracture prevention is important for maintaining function
   - Contracture prevention is important for the prevention of pain/stiffness
   - Contracture prevention is important for a good sitting posture and activities of daily living (ADL)
   - Contracture prevention is important for a patient's appearance
   - Contracture prevention is carried out at the request of child/parent
   - Contracture prevention is important to avoid surgical intervention

Thoughts on starting/stopping and type of contracture treatment

1. I initiate therapy as part of the management of contractures: (multiple answers possible)

*Before a contracture can develop, as soon as a contracture arises, as soon as a contracture worsens, as soon as a contracture gives rise to complaints, that depends on the joint, that depends on the treatment of other joints which has already started, otherwise:*

1. If I recommend starting to use a standing table, then preferably at the age:

*0-1 years, 1-2 years, 2-5 years, >5 years*

*Open text field: which conditions (of the child or external) do you consider necessary for starting to use a standing table?:*

1. Can you indicate for what reasons you would stop the treatment of contractures?

*Note (required field):*

**Part 4:** **Contracture management, prevention/treatment in relation to guidelines**

Dutch guideline SMA type 1

The following statements are based on the advice regarding contracture prevention from the guideline on Spinal muscular atrophy (SMA) type 1 ([Startpagina - SMA type 1 - Richtlijn - Richtlijnendatabase](https://richtlijnendatabase.nl/richtlijn/spinale_musculaire_atrofie_sma_type_1/startpagina_-_sma_type_1.html)).

For the following patient groups, regardless of whether or not you treat a child in this specific target group, could you indicate the extent to which you agree with the statement? *1=strongly agree, 2=agree, 3=disagree, 4=strongly disagree*

Please give answers for three different patient severity levels:

1. **Presymptomatically treated group**: children who started with DMT prior to the onset of clinically manifest symptoms.
2. **Symptomatically treated, group 1**: children who initiated DMT within two years after diagnosis.
3. **Symptomatically treated, group 2**: children who started with DMT more than two years after diagnosis.

*Stretch the muscles and maintain length, within the pain threshold, at least three to five times a week, sustaining the final position for at least thirty seconds.*

Proposition 1:

I advise parents to stretch muscles fully and maintain them in accordance with the above statement.

• Group 1:

• Group 2:

• Group 3:

*Open text field for additional comments (optional)*

Proposition 2: *Combat contractures by performing prolonged stretching. Static and dynamic orthoses can be used for this, if indicated.*

Basically, I apply static and/or dynamic orthoses in this target group.

• Group 1:

• Group 2:

• Group 3:

*Open text field for additional comments (optional)*

Proposition 3: *Consider using a corset for children with SMA type 1C or children who are receiving medication if postural ketosis is present.*

I am considering using a corset for children with SMA type 1C or children who are being treated with medication if postural glucose is present.

• Group 1:

• Group 2:

• Group 3:

*Open text field for additional comments (optional)*

International Standards of Care

The following statements are based on the recommendations on contracture prevention for children with SMA types 1, 2 and 3 from the Standards of Care for SMA: *Diagnosis and management of spinal muscular atrophy: Part 1: Recommendations for diagnosis, rehabilitation, orthopedic and nutritional care by Mercuri et al 2018* ([Diagnosis and management of spinal muscular atrophy: Part 1: Recommendations for diagnosis, rehabilitation, orthopedic and nutritional care - ScienceDirect](https://www.sciencedirect.com/science/article/pii/S0960896617312841?via%3Dihub).)).

For the following patient groups, regardless of whether or not you treat a child in this specific target group, could you indicate to what extent you agree with the statement? *1=strongly agree, 2=agree, 3=disagree, 4=strongly disagree*

Please give answers for three different patient severity levels:

1. **Presymptomatically treated group**: children who started with DMT prior to the onset of clinically manifest symptoms.
2. **Symptomatically treated, group 1**: children who initiated DMT within two years after diagnosis.
3. **Symptomatically treated, group 2**: children who started with DMT more than two years after diagnosis.

**Non-sitters**

*Stretching: Daily use of upper lower limb orthoses for stretching and to promote function and range of motion. Static orthoses, knee immobilizers and hand splints are recommended for positioning and stretching. AFOs and KAFOs can be used for stretching and positioning. TLSOs (thoraco-lumbo-sacral orthosis) are used for positioning. Supported standing.*

*The minimal frequency for stretching and range of motion is 3–5 times per week. The minimal frequency for bracing to be effective is 5 times per week.*

Proposition 1: In children who cannot sit independently, I recommend a minimum frequency for stretching of 3-5 times a week.

• Group 1:

• Group 2:

• Group 3:

*Open text field for additional comments (optional)*

Proposition 2: For children who cannot sit independently, I recommend daily use of hand splints for range of motion and functioning.

• Group 1:

• Group 2:

• Group 3:

*Open text field for additional comments (optional)*

Proposition 3: For children who cannot sit independently, I recommend daily use of AFOs and/or KAFOs for range of motion.

• Group 1:

• Group 2:

• Group 3:

*Open text field for additional comments (optional)*

Proposition 4: For children who cannot sit independently, I recommend daily use of TLSO for the purpose of positioning.

• Group 1:

• Group 2:

• Group 3:

*Open text field for additional comments (optional)*

**Sitters**

Stretching: Orthoses are used for the upper and lower limbs to promote function and ROM. Regular stretching for segments known to be at risk of contractures: hip, knee and ankle, wrist and hand. Knee immobilizers, KAFOs, and AFOs are recommended for positioning and standing. RGOs (Reciprocating Gait Orthosis) and KAFOs can be used for supported ambulation. TLSOs and hand splints are used for positioning.

*Minimal frequency for stretching and ROM: 5–7 times/week. When stretching or performing joint mobilization, ensure joint segments are aligned throughout the treatment. Supported standing should be up to 60 minutes and minimal frequency is 3–5 times/week, optimal 5–7 times/week.*

Proposition 3: In children who can sit independently, I recommend a minimum frequency of stretching to maintain ROM of 5-7 times a week.

• Group 1:

• Group 2:

• Group 3:

*Open text field for additional comments (optional)*

Proposition 4: In children who can sit independently, I recommend using a standing table for a maximum of 60 minutes, with a minimum frequency of 3-5x per week and an optimal frequency of 5-7x per week.

• Group 1:

• Group 2:

• Group 3:

*Open text field for additional comments (optional)*

**Ambulant**

*Stretching: minimal frequency: 2–3 times/week, optimal: 3–5. Maintain flexibility through active assisted stretching and include the use of orthoses according to specific needs.*

Proposition 5: In ambulatory children I recommend actively stretching 2-3 times a week (with an optimal frequency of 3-5x per week).

• Group 1:

• Group 2:

• Group 3:

*Open text field for additional comments (optional)*

Are there any aspects related to contracture treatment that have not been highlighted in the above survey that you think are important to share with us? {Open text field:}

If you are willing to be approached at a later date for in-depth questions about this survey, would you please enter your name and e-mail address and/or telephone number here?

{Open text field:}
